# AFM-IR Depth Sensitivity on Vertically Stratified Polymer Structures

**DOI:** 10.1021/acs.analchem.6c01324

**Published:** 2026-09-07

**Authors:** Devon S. Jakob, Jeffrey J. Schwartz, Karen E. Grutter, Andrea Centrone

**Affiliations:** † Nanoscale Device Characterization Division, Physical Measurement Laboratory, 10833 National Institute of Standards and Technology, Gaithersburg, Maryland 20899, United States; ‡ Laboratory for Physical Sciences, College Park, Maryland 20740, United States; § Department of Electrical and Computer Engineering, University of Maryland, College Park, Maryland 20742, United States

## Abstract

By transducing a sample’s photothermal expansion and contraction, the photothermal induced resonance technique (PTIR, also known as AFM-IR) enables characterization of light absorption at scales (≈10 nm) well below the limits imposed by far-field diffraction. However, AFM-IR depth sensitivity on heterogeneous samples remains poorly understood, making quantitative analyses challenging. Here, we examine stratified, three-dimensionally printed, polymer heterostructures engineered to elucidate the AFM-IR signal dependence on detection frequency, light polarization, and measurement modality (i.e., on- or off-resonance). Results show that the signal intensity, derived from absorption in the topmost layer (here, an epoxy-based prism), increases nearly linearly with its thickness regardless of detection frequency or resonance condition, aligning with previous observations in homogeneous (non-stratified) samples. However, the signal from the bottom layer (here, an acrylic photopolymer resin disk beneath the epoxy prism) can be amplified, attenuated, or remain unchanged, depending on the detection frequency and measurement modality. On-resonance, the signal coming from absorption in the bottom, acrylic layer always decays approximately exponentially with the thickness of the top, epoxy layer, with a frequency-dependent decay rate. Notably, absorption from the acrylic layer was detected even beneath the thickest epoxy region (here, ≈440 nm) for all measurement conditions, highlighting the considerable subsurface sensitivity of AFM-IR. We propose adopting a ≈95% signal decay as a convention to describe a practical AFM-IR measurement depth sensitivity (probed depth), as opposed to the previously suggested ≈63% decay. This work advances AFM-IR-based nanoscale chemical imaging by elucidating the relationship between signal attenuation and sample stratification, which represents a critical step toward more-quantitative and three-dimensional compositional analyses.

## Graphical abstract

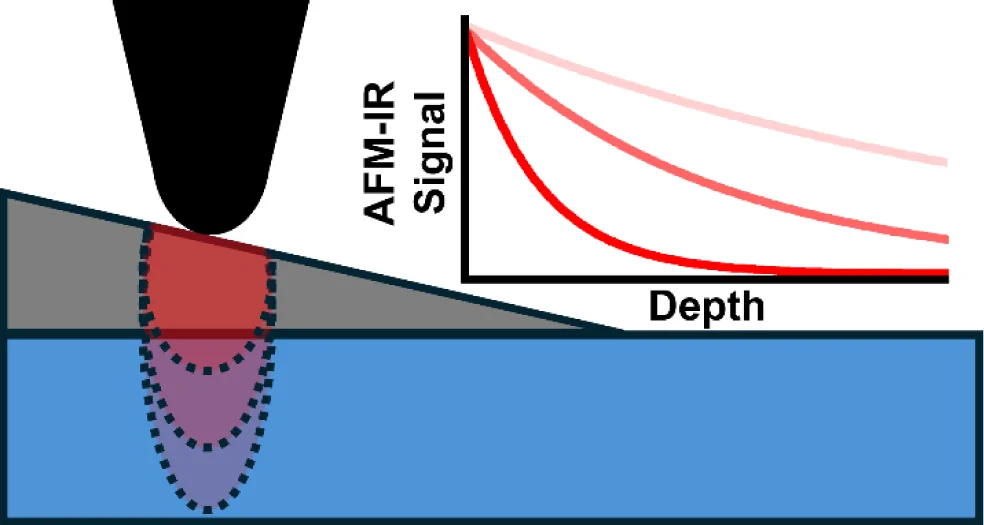

The AFM-IR^1–7^ technique uses the probe tip of an atomic force microscope (AFM) to measure absorption spectra and maps with lateral resolution down to ≈10 nm.^8^ Such spectra typically have undistorted, absorptive profiles that correspond closely with far-field infrared (IR) spectra.^9,10^ These characteristics arise from the mechanical transduction of the sample photothermal expansion by the AFM probe,^11–13^ which is directly proportional to the sample’s local optical absorption coefficient.^9,14,15^ Since AFM-IR has been extended into the visible and near-IR ranges, it is also sometimes more generally referred to as photothermal induced resonance (PTIR) to highlight the detection mechanism rather than the spectral range used to excite the sample. In the mid-IR, it enables the identification of chemical groups,^16,17^ materials,^18–20^ polymorphs,^21^ stacking order in graphene,^22^ as well as the imaging of sub-diffraction excitations such as polaritons^23^ and plasmons^24,25^ In the visible and near-IR, PTIR characterizes and maps electronic transitions,^8,26^ bandgaps,^27^ and defects^20,27^ at the nanoscale. These useful capabilities contribute to the growing popularity and application of PTIR in diverse fields such as medicine,^28–33^ photonics,^23^ materials science,^18–20,34,35^ geology,^36,37^ art conservation,^16^ and photovoltaics,^38^ among others.^1–4^

Distinctively, AFM-IR can sense sample compositions at depths exceeding 1 μm.^4,10,15^ For example, the AFM-IR signal intensity was reported to increase nearly linearly with sample thickness on a ≈2.5 μm thick polymer cone with top illumination.^10^ For total internal reflection (TIR) illumination, an approximately linear signal dependence was reported for a poly­(methyl methacrylate) (PMMA) wedge up to ≈1 μm thick.^9,15^ The compositions of several heterogeneously stratified samples have also been characterized with AFM-IR. For example, polydimethylsiloxane residue was detected within 2D materials heterostructures^19^ and under crystals,^23^ while polyurethane was detected under ≈200 nm thick bacteria.^39^ Numerous other examples have been reported in biological applications, including localizing viruses inside bacteria,^40^ drug molecules inside liposomes,^33^ polymeric nanoparticles inside cells,^41^ and protein aggregates inside cells^42^ and fungi.^43^ Such sensitivity to compositions far below the sample surface contrasts with other scanning-probe methods, such as tip-enhanced Raman scattering^3^ (exclusively surface sensitive) and scattering-type scanning near-field optical microscopy^1^ (near-surface sensitive, ≈25 nm–100 nm).

However, quantitative compositional and multivariate analyses^43,44^ using AFM-IR are challenging since the signal intensity depends on the local properties of the sample (e.g., absorption and expansion coefficients, stiffness, mechanical damping, etc.) and on the frequency-dependent transduction efficiency of the AFM probe.^4,45^ Both theoretical and experimental studies have elucidated AFM-IR signal transduction,^9,14,15,45–47^ with efforts made to obtain measurements with reduced dependences on the sample mechanical properties.^31,39^ The AFM-IR signal originates from the absorption of light by the sample. Its subsequent photothermal expansion and contraction excites mechanical oscillations in the AFM cantilever. Additionally, the wide-bandwidth sensitivity of specialized optomechanical probes^11–13^ have recently enabled time-resolved measurements of the sample’s photothermal expansion and contraction directly. The characteristic time (*τ*) over which the sample thermalizes with its surroundings critically affects the signal transduction amplitude. This time scale, and its relationship with other material properties, is described by eq 1,^12^

1 $$z=\sqrt{\frac{\tau \eta }{\rho c}}\tan^{-1}\left(\frac{G}{\eta }\sqrt{\frac{\tau \eta }{\rho c}}\right)$$

where *η* is thermal conductivity, *ρ* is the density, *c* the specific heat capacity, and *z* is the thickness of the sample, and *G* the sample-substrate interfacial thermal conductance.^11,12^ As discussed in previous work,^11,12^ eq 1 is a good approximation for any sample domain that (i) is homogeneously photothermally heated (i.e., optically thin) and (ii) subsequently thermalizes via one-dimensional (vertical) diffusion to a heat sink substrate. In the limit of infinite *G* (i.e., no thermal boundary resistance, denoted here as *G*_∞_), or in the limit of thick samples (*z* ≫ *l*_0_ = *η*/*G*), eq 1 simplifies to^11,12^

2 $$\tau =\frac{4\rho cz^{2}}{\pi^{2}\eta }$$

Recently, we developed a discrete Fourier transform (DFT) theory for AFM-IR signal transduction^10,45^ that links the time-domain expansion and contraction of the sample to the AFM cantilever excitation amplitudes, in the frequency domain. This excitation depends on the laser parameters (pulse length, pulse shape, repetition rate), cantilever detection frequency, and *τ*. The model predicts an improvement in AFM-IR lateral resolution with detection frequency and for lower *G*, independent of the laser pulse length.^45^ Furthermore, the model predicts a nearly linear signal increase with sample thickness and a decrease of signal sensitivity with detection frequency, all in good agreement with experimental data on homogeneous samples and s-polarized illumination.^10,45^

Just a few recent studies have directed their attention toward vertically heterogeneous, stratified samples, which remain a not well understood frontier of AFM-IR analysis. In TIR illumination, calculations for a 1 nm thick weakly absorbing layer embedded inside a 1 μm thick non-absorbing matrix indicate a decreasing signal intensity as a function of the layer depth for both s- and p-polarizations.^9^ More recently, a Green’s function-based thermoelastic model has been proposed to assess AFM-IR resolution and signal intensity for a ≈140 nm diameter PMMA particle embedded within a non-absorbing polyethylene film (≈1 μm thick) as a function of detection frequency and depth.^46^ In agreement with previous investigations,^45^ this thermoelastic model and experiment (s-polarization) confirms that the AFM-IR spatial resolution improves with detection frequency. The model also predicts a decreased spatial resolution and signal intensity from particles buried more deeply within a matrix.^46^ Importantly, this finding poses a challenge for the quantification of chemical composition in stratified samples but also provides an opportunity for reconstructing the sample stratification with AFM-IR. A first attempt of such reconstruction using AFM-IR maps (p-polarization) at several different frequencies was reported for polystyrene (PS) particles within a PMMA matrix.^47^ The sample’s three-dimensional reconstruction was inferred based on a material-specific, multifrequency calibration on a stratified sample consisting of a PS wedge on a PMMA film (≈18 μm thick) on a Si substrate, which revealed shallower probing of the sample composition for increasing detection frequencies. However, despite recent progress, a knowledge gap remains on the AFM-IR probed depth and in the quantitative analysis of AFM-IR data for vertically heterogeneous, stratified samples. To better isolate and understand the critical parameters affecting AFM-IR signal intensity and probed depth in a stratified system, we crafted an heterogeneous polymer sample composed of an IP-Dip2[Fn ba-fn1] disk partially covered by an SU-8[Fn ba-fn1] triangular prism. To rationalize the many competing effects, we examine the signal dependence under a broad range of experimental conditions. For example, we use both off-resonance^10^ and resonance-enhanced^33,48^ cantilever excitation schemes to avoid complications introduced by resonance tracking and to consider the most common experimental conditions, respectively. We use both s-polarized and p-polarized excitations, to minimize and exploit enhancement effects from the AFM tip near-field. Absorption in the top (SU-8) layer increases nearly monotonically, and nearly linearly, with increasing SU-8 thickness while using both on- and off-resonance detection schemes, independent of frequency. This result agrees with previous measurements on homogeneous, non-stratified, samples.^10,15,49^ For off-resonance excitations, the AFM-IR signal intensity due to absorption in the bottom (IP-Dip2) layer can be both amplified or reduced with respect to bare IP-Dip2 depending on the cantilever detection frequency and tip-sample interactions. Therefore, measuring the true composition of the bottom layer in stratified samples off-resonance requires selecting an *ad hoc* detection frequency that minimizes the effects of near-surface tip-sample interactions across the sample. In contrast, for on-resonance excitations, the AFM-IR signal intensity due to absorption in the bottom (IP-Dip2) layer is attenuated by the SU-8 top layer for all the measured frequencies independent of polarization. Notably, IP-Dip2 absorption in the nearly uniformly thick layer under the SU-8 prism is detected in all images, suggesting a probed depth >440 nm (the maximum SU-8 thickness) for all the frequencies tested (up to 1545 kHz). As suggested by Dazzi et al.,^47^ we fit a decaying exponential function to the signal attenuation profile as a function of SU-8 thickness. While we considered other effects that could affect the signal intensities, those are found to be either small, thickness-independent (e.g., refractive index contrast), or predict effects contrary to the experimental findings (e.g., variations in mechanical *Q*-factors), which we rule out as possible explanations. One main conclusion of this work is that the probed depth of AFM-IR is not an intrinsic characteristic of the measurement parameters (e.g., the repetition rate) but depends on the extent which heat accumulates in the sample, i.e., on the interplay between the laser pulse spacing and the sample thermalization time. By highlighting the effects of sample stratification on the AFM-IR signal intensities, this work bolsters nanoscale chemical imaging and facilitates more quantitative analyses by aiding efforts to reconstruct the composition of stratified samples.

## Methods

### Sample Preparation

Similar to our previous study,^10^ we used a commercially available two-photon polymerization^50^ patterning tool to fabricate the structures measured in this work. First, IP-Dip2 disks were patterned on the surface of an IR-transparent BaF_2_ optical window. During patterning, a high numerical aperture objective was immersed in the unpolymerized liquid resin while a pulsed, near-IR laser (center wavelength 780 nm) illuminated the sample. Only the light focused within a small, three-dimensional region (voxel) is sufficiently intense to induce polymerization of the photocurable resin via two-photon absorption. Three-dimensional optical patterning is achieved by moving the voxel location relative to the sample to polymerize the resin in the desired form. Unpolymerized resin was later removed by immersing the sample in propylene glycol monomethyl ether acetate (≈30 min), followed by rinsing with isopropanol and blowing dry with N_2_. Afterward, to create the SU-8 wedges, SU-8 2015 was spun (4000 rotations/min) onto the IP-Dip2-patterned sample and baked at 90 °C for 1 min. Then, a glass coverslip was placed on top of the SU-8 layer, with care exercised to avoid trapping air bubbles, and baked for a second time (180 °C for 4 min). Patterning of the SU-8 structures proceeded similarly to the process described with IP-Dip2 with an exception that immersion oil (refractive index ≈ 1.5980) filled the gap between the objective and the glass interface.

### PTIR Measurements

Off-resonance AFM-IR measurements were obtained in contact mode using a modified, commercially available instrument described previously,^8^ a QCL with wavelength tunable from 910 cm^–1^ to 1905 cm^–1^, and gold-coated silicon AFM probes (nominal 0.07 N/m–0.4 N/m spring constant and a free-space oscillation frequency of 13 kHz ± 4 kHz). In all measurements the AFM cantilever was oriented with its long axis parallel to the SU-8 prism apex. A parabolic mirror focused the laser light at a ≈20° oblique incident angle with respect to the sample surface to a spot of ≈50 μm in diameter, centered around the probe tip. A pair of precisely calibrated beam-steering mirrors maintained the laser spot position relative to the AFM probe at all wavelengths, correcting for any wavelength-dependent beam-pointing errors. Spectral intensities were computed as the ratios of the measured AFM-IR signal to the laser output intensity (background spectrum) measured with a pyroelectric detector with identical experimental conditions (i.e., laser pulse length, repetition rate). The laser light was s-polarized, with the electric field oriented parallel in the sample plane (perpendicular to the longitudinal axis of the probe tip). Absorption maps were obtained by raster scanning the AFM probe over the sampled region while illuminating the sample at a constant wavelength. Spectra were obtained by holding the probe stationary while sweeping the laser wavelength between 920 cm^–1^ and 1890 cm^–1^ at a rate of 50 cm^–1^/s and with a resolution of 1 cm^–1^. Fast-Fourier transforms of the cantilever signal were used to extract the AFM-IR signal amplitude at the frequency corresponding to the laser excitation frequency using a ±50 kHz bandpass filter centered on the laser repetition frequency. At each location, 10 spectra were averaged, and a Savitzky–Golay filter with a third-order polynomial to smooth the averaged spectra through a least-squares approximation.

Resonance-enhanced AFM-IR images were obtained like the off-resonance images, except for the laser repetition rate. The repetition rate was selected to correspond with the mechanical resonance frequencies of the AFM cantilever (e.g., ≈190 kHz, ≈391 kHz, ≈1145 kHz, and ≈1545 kHz), determined by sweeping the laser repetition rate while the tip is placed stationary on a portion of SU-8 that covers IP-Dip2 and monitoring the cantilever response. A phase-locked loop, with integral and proportional gains of 5 and 10, respectively, was employed to compensate for changes to this resonant condition resulting from local sample mechanical properties.

### Data Processing

Topography and AFM-IR absorption images were measured simultaneously by scanning the probe across the sample at a rate of 0.2 Hz. Typically, absorption maps (i.e., absorption as a function of position) are mostly independent of the sample topography and plane tilt. Here, however, we process both, nominally independent, data channels to relate signal variations as a function SU-8 thickness. By first scanning large areas of the sample to include the BaF_2_ substrate on opposite sides of the SU-8/IP-Dip2 polymer heterostructure, we determined the overall tilt plane of the topography image. Once known, SU-8 wedge thicknesses can be estimated from smaller, higher resolution, plane-corrected topography maps of parts of the polymer heterostructure.

The SU-8 edge location (where it has zero thickness) was identified by finding the position at which topographic height *versus* position and absorption *versus* position profiles both plateau to a near-constant value. This point denotes the minimum-thickness (*z* = 0) bound in the data, beyond which (i.e., on bare IP-Dip2) is not considered in further analysis. Within this bound (i.e., *z* > 0), the height measurements are unevenly distributed due to the rough texture of the SU-8 wedge. To obtain a consistent, uniform sampling over the height of the SU-8 wedge, we averaged measurements within narrow, uniformly distributed height ranges (1 nm). Finally, an exponentially decaying function (eq 3) is fit to these profiles to determine the best estimates of the three fitting parameters (*m*_1_, *m*_2_, and *P*_depth_). Reported errors are derived from least-squares fit covariance matrices.

## Results and Discussion

Here, we analyze a stratified SU-8/IP-Dip2 polymer heterostructure fabricated on a polished BaF_2_ substrate. This structure consists of an IP-Dip2 disk (≈50 μm diameter and ≈320 nm thick), patterned directly on the BaF_2_ substrate, partially covered by an asymmetric SU-8[Fn ba-fn1] triangular prism (extending up to ≈760 nm above the substrate and up to ≈440 nm over the IP-Dip2 disk); see Figure 1a–c. We note that the fabrication process created a raised lip that protrudes vertically (≈105 nm) at the IP-Dip2 disk periphery (Figure 1c). However, for most of this analysis, we focus on the region of the sample with an approximately linearly varying SU-8 wedge atop a nearly uniform IP-Dip2 disk.

**Figure 1:**
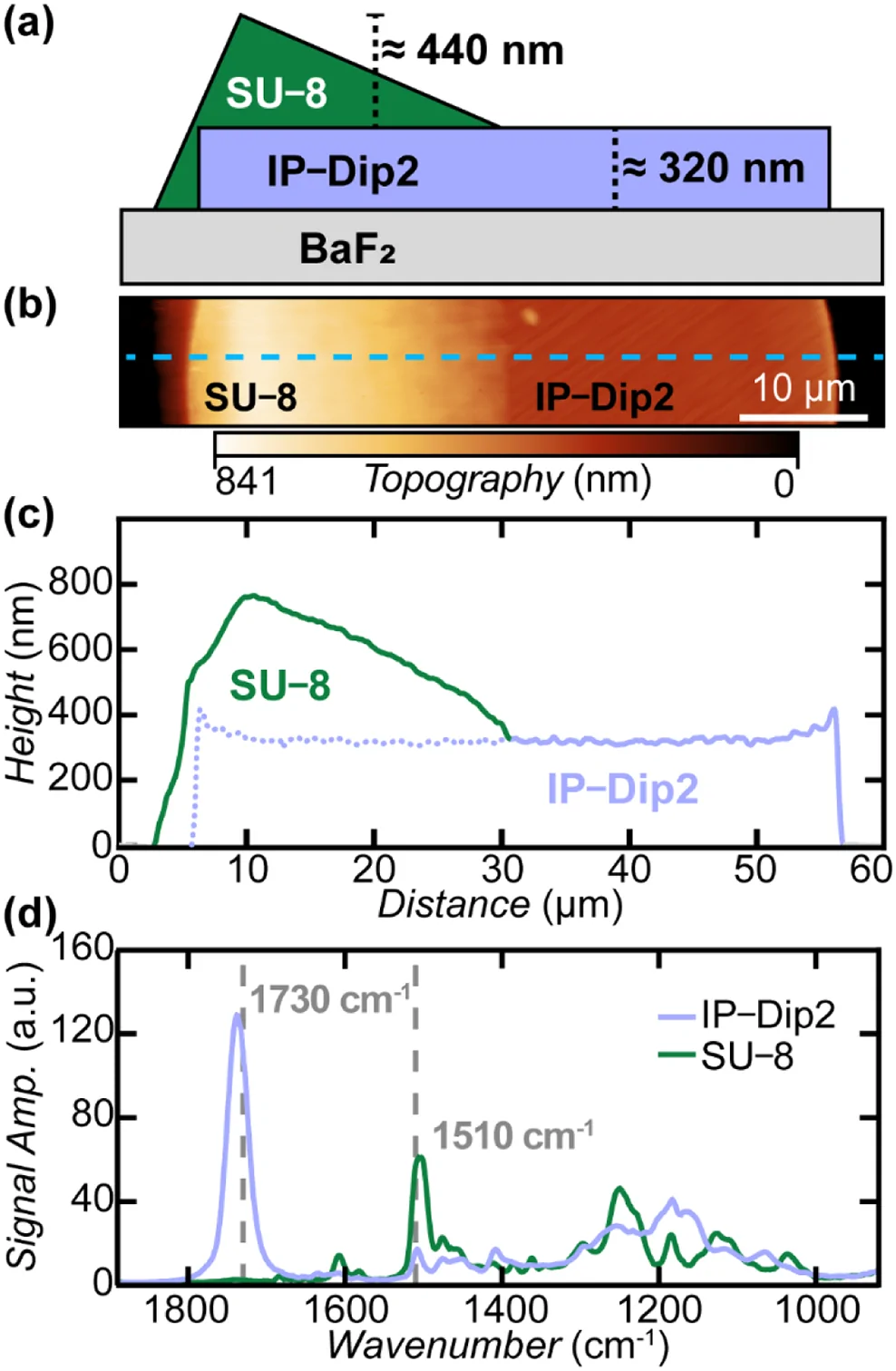
(a) Schematic of the stratified polymer sample, consisting of an IP-Dip2 disk (≈50 μm diameter and ≈320 nm thick) partially covered by an asymmetric SU-8 triangular prism (up to ≈440 nm thick), fabricated on a BaF_2_ substrate. (b) Topography map and (c) line profile of the sample along the dashed blue line in (b). The left side of the IP-Dip2 disk is covered by the SU-8 prism (green profile) with a thickness that varies approximately linearly away from its apex. By contrast, the right side of the IP-Dip2 disk is exposed, revealing a relatively flat and uniformly thick profile (in blue). The left portion of the disk profile, dashed line beneath the prism and not directly measured, is shown mirrored from the right side and is provided to guide the eye. (d) Representative absorption spectra (s-polarization) showing characteristic absorption peaks for SU-8 (green, ≈1510 cm^–1^) and IP-Dip2 (blue, ≈1730 cm^–1^).

This structure has useful characteristics for assessing the effects of the sample stratification on AFM-IR depth sensitivity. First, the two polymers have distinct characteristic IR absorption peaks (e.g., ≈1510 cm^–1^ for SU-8 and ≈1730 cm^–1^ for IP-Dip2; see Figure 1d), which enables control over where (in which layer) the photothermal signal originates. Second, the small aspect ratio of the structure and large IR laser spot size (≈50 μm) ensure, in good approximation, one-dimensional heat diffusion toward the substrate within the photothermally heated sample,^12^ which is well described by eq 1. Third, the full polymer structure and part of the substrate can be imaged within a single scan window, enabling accurate plane tilt correction (typical of AFM) and providing a reliable baseline for determining polymer thicknesses. Fourth, the variable thickness of the SU-8 prism (up to ≈440 nm, ≈1.14° slope on the long side) covering the IP-Dip2 disk enables assessment of the effects of the non-absorbing top layer on the transduced AFM-IR signal intensity stemming from light absorption in the bottom layer. However, we note that the surface roughness of the 3D printed structures is not ideal for estimating the local sample thickness of the bottom (IP-Dip2) and top (SU-8) layers and can also make resonance tracking in AFM-IR experiments challenging.

In resonance-enhanced AFM-IR measurements, an external cavity quantum cascade laser (QCL) with tunable repetition rate, like the laser employed here, is typically used to excite oscillations of one of the mechanical modes of the AFM cantilever.^48^ By matching the laser pulse rate to one of the cantilever mechanical modes, the signal amplitude is resonantly amplified by ≈*Q*/2π, where *Q* is the quality factor of the selected cantilever mode.^48^ Although this method typically leads to higher signals and measurement throughputs,^4^ the resonance gain also depends on the local mechanical properties of the sample, such as its stiffness (causing resonance shifts) and damping (causing variation in *Q* and the signal gain).^4,31^ While changes to the resonant condition due to frequency shifts are typically compensated with a phase-locked loop (PLL),^33^ variations of *Q* remain a challenge. Therefore, here we use both on- and off-resonance^10^ AFM-IR detection schemes, which excite samples (and detect signals), at frequencies that match, or intentionally mismatch, cantilever mechanical resonances, respectively.

For simplicity, we start with off-resonance measurements. At 1510 cm^–1^, AFM-IR maps primarily show light absorption in the SU-8 prism (top layer in stratified heterostructure). These maps display a nearly linear monotonic increase in signal intensity with increasing SU-8 thickness at all cantilever detection frequencies (see Figure 2a–d and Figure S1). This result is in good agreement with previous measurements on homogeneous samples (i.e., consisting of a single material with variable thickness on top of a non-absorbing substrate).^10,15,49^ In first approximation, and in absence of material-specific mechanical, thermal, elastic, optical, and acoustic contributions to the signal intensity,^51^ the AFM-IR signal generated by the bottom IP-Dip2 disk should also be constant since the nearly constant-thickness layer would homogeneously absorb and expand regardless of the SU-8 layer thickness. In practice, however, this assumption does not hold true, as evident when comparing AFM-IR maps and their intensity profiles at 1730 cm^–1^ (characteristically absorbed by IP-Dip2 but not SU-8) obtained at different laser repetition rates (*f*_pulse_) and at constant duty cycle (≈10%); see Figure 2e–h and Figure S2. Surprisingly, the map at 100 kHz (Figure 2e) shows a slightly stronger signal in the IP-Dip2 region covered by SU-8 compared to the bare IP-Dip2 region. This observation appears somewhat analogous to previously reported measurements of dried bacteria deposited on a polyurethane film. There, a stronger polyurethane signal was attributed to differences in mechanical properties, which resulted in larger mechanical *Q* factors when the tip was positioned on the top (bacterial) layer.^39^ Furthermore, here, the IP-Dip2 signal intensity in the covered region increases with SU-8 thickness (Figure 2e, h). In contrast, at 714 kHz (still off-resonance) the signal intensity of the bare IP-Dip2 region is about 3 × stronger than in the SU-8 covered region (Figure 2g, h), suggesting a higher surface sensitivity at this frequency or an attenuation effect by the SU-8 layer. At an intermediate frequency, 302 kHz, the AFM-IR intensity is approximately constant across the entire IP-DiP2 region (Figure 2f, h), thus providing a more accurate representation of the sample composition and structure. Interestingly, all the images obtained at 1730 cm^–1^ (Figure 3e–g) reveal the presence of the raised rim near the edge of the IP-Dip2 disk, even beneath the SU-8 prism, confirming the utility of the AFM-IR technique for detecting and identifying subsurface composition, at least qualitatively. The variation of the signal intensity at 1730 cm^–1^ across the structure (i.e., the contrast obtained) is attributed to local variations of tip-sample mechanical interactions and to the cantilever transduction efficiency as a function of the local mechanical properties (see Figure S3), which are significantly reduced at 302 kHz. This finding suggests that the laser repetition rate for off-resonance AFM-IR measurements on heterogeneous or stratified samples could, in principle, be selected *ad hoc* to minimize the effect of the mechanical properties on the tip-sample interactions and therefore on the AFM-IR intensity maps, as suggested in a previous study.^52^ We note, however, that the frequency (or frequencies) achieving such transduction neutrality is not an intrinsic property of the sample but rather are a function of the tip-sample interactions and will be different when using another AFM probe due to probe-to-probe variability of mechanical properties. For example, by using a different AFM probe, we obtained an approximately constant-intensity image of the IP-Dip2 disk at 477 kHz (see Figure S4). Although this approach works reasonably well for a bi-material system, it could be difficult to implement for samples containing several different materials/phases.

**Figure 2:**
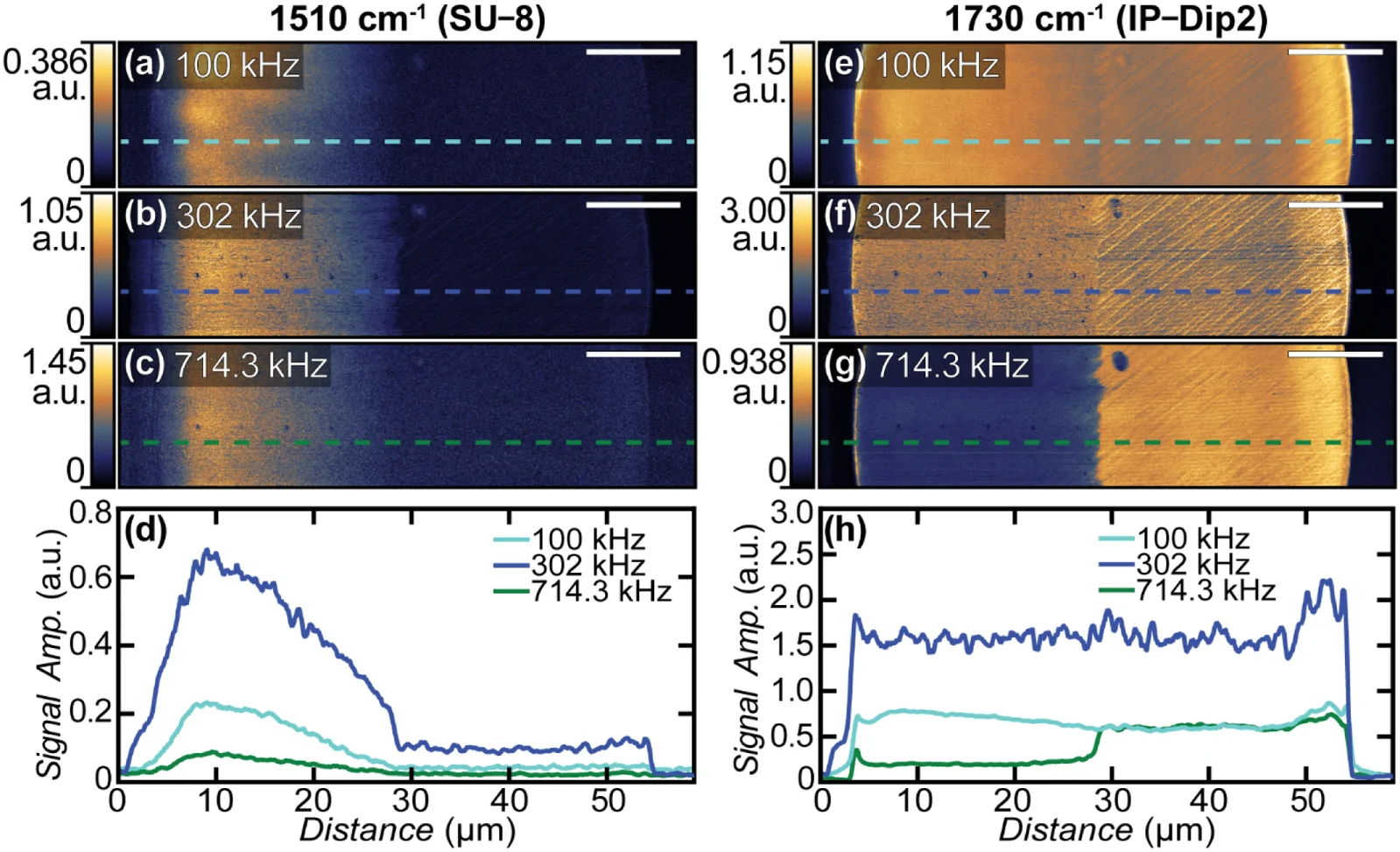
Off-resonance AFM-IR absorption maps of a sample consisting of an IP-Dip2 disk partially covered by an SU-8 asymmetric triangular prism at 1510 cm^–1^ (a–c) and 1730 cm^–1^ (e–g), corresponding to absorption primarily by SU-8 and IP-Dip2, respectively. Measurements were obtained at different laser repetition rates: 100 kHz (a, e), 302 kHz (b, f), and 714.3 kHz (c, g). Intensity profiles at (d) 1510 cm^–1^ and (h) 1730 cm^–1^ along the color-coded lines in (a–c) and (e–g), respectively. All measurements used a ≈10% laser duty cycle with s-polarization. Scale bars are 10 μm.

**Figure 3:**
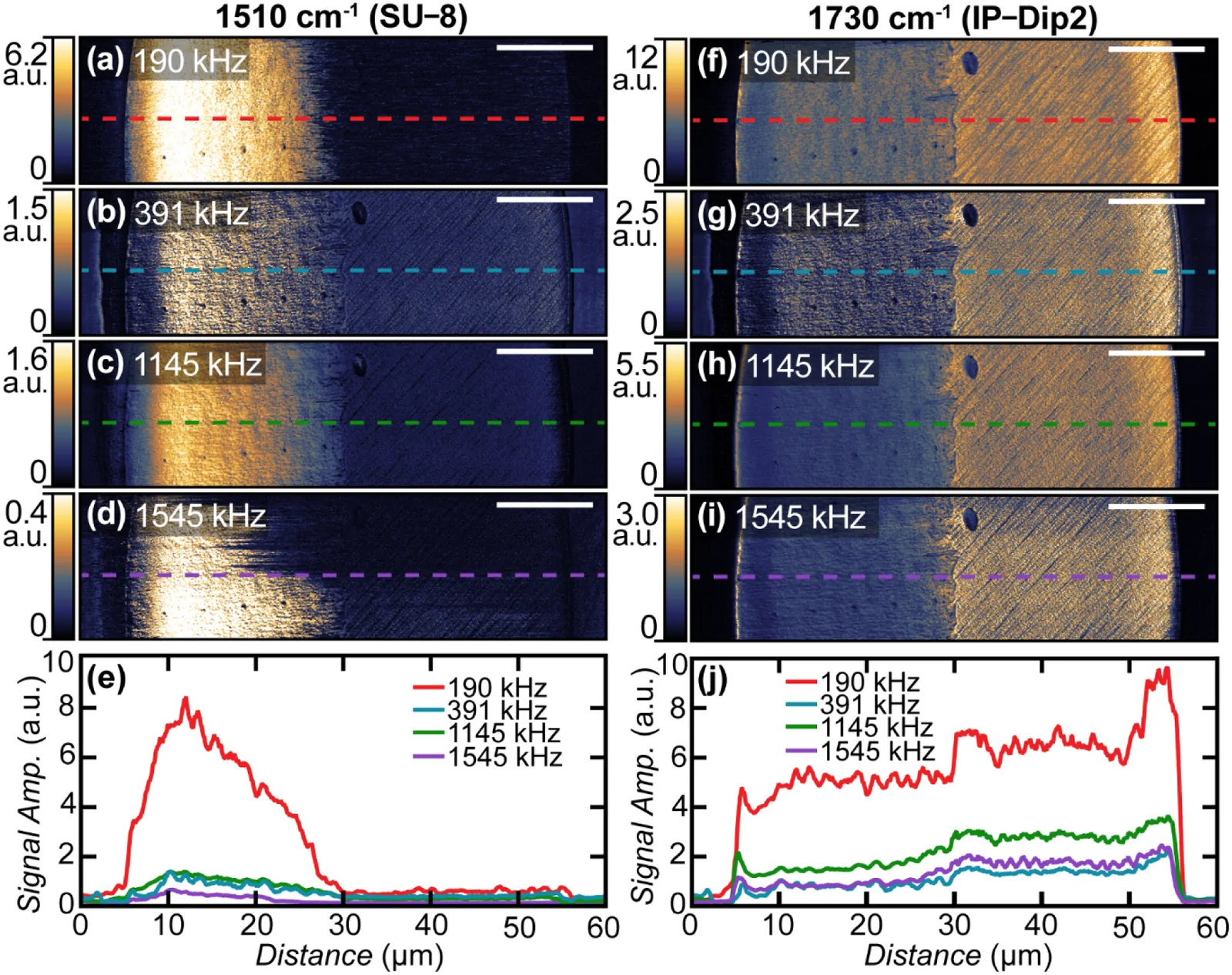
Resonance-enhanced AFM-IR absorption maps of a sample consisting of an IP-Dip2 disk partially covered by an SU-8 asymmetric triangular prism at 1510 cm^–1^ (a–d) and 1730 cm^–1^ (f–i), corresponding to absorption primarily by SU-8 and IP-Dip2, respectively, measured at different laser repetition rates. Intensity profiles at 1510 cm^–1^ (e) and 1730 cm^–1^ (j), along the color-coded lines in (a–d) and (f–i), respectively, are shown using a common intensity scale. All measurements were obtained with a ≈10% laser duty cycle with s-polarization. All scale bars are 10 μm.

Next, we analyze the same SU-8 prism/IP-Dip2 disk structure using the more commonly employed resonance-enhanced (RE) excitation scheme, which typically provides higher signal-to-noise ratios and throughputs. In RE-AFM-IR, the laser pulse frequency is matched to one of the contact resonant frequencies of the AFM cantilever (here, ≈190 kHz, ≈391 kHz, ≈1145 kHz, and ≈1545 kHz); see Figure 3. A PLL compensates for small shifts in the cantilever mechanical resonances and maintains the resonant excitation condition throughout the measurements.^33^ We note that the PLL cannot compensate variations of *Q* (affecting the mechanical gain). Images obtained at 1510 cm^–1^, primarily corresponding to SU-8 absorption, once again display a signal that increases monotonically with the SU-8 thickness (Figure 3a–e), analogous to wedge-like samples made by a single material.^10,15,42^ By contrast, for all the measured frequencies, the resonance-enhanced images of the IP-Dip2 disk (1730 cm^–1^) clearly show a stronger signal measured on the bare IP-Dip2 region of the sample, compared with region covered by the SU-8 prism (Figure 3f–j). In particular, the IP-Dip2 signal drops abruptly at the edge with the SU-8 prism. This suggests that the top SU-8 layer somehow attenuates the AFM-IR signal originating from absorption in the buried IP-Dip2 layer. Notably, the IP-Dip2 disk’s protruding rim (estimated ≈105 nm thicker than the disk, based on the exposed topography) is detected below the SU-8 prism in all images. The IP-Dip2 rim is estimated to be only about 30 nm to 40 nm below the SU-8 surface. However, IP-Dip2 absorption in the flat region of the disk is also detected in all images, suggesting a depth sensitivity >440 nm (i.e., the maximum SU-8 thickness) for all the frequencies tested.

Since the IP-Dip2 absorption maps in Figure 3f–i show abrupt discontinuities at the edge of the SU-8 prism, we remeasured the central portion of the sample, across this edge, with higher pixel resolutions (23.9 nm in Figure 4a–d, and 9.8 nm in Figure 4f–i) to gain more detailed insights. For these additional measurements, we used p-polarization to enable a more direct comparison with literature data on a similarly structured sample.^47^ From the real-space images in Figure 4, we extract the IP-Dip2 absorption signal intensity as a function of the SU-8 overlayer thickness, shown in Figure 4e, j. In these one-dimensional representations, the signal decay appears less abrupt than in the real-space images; see also the s-polarization images in Figure S5.

**Figure 4:**
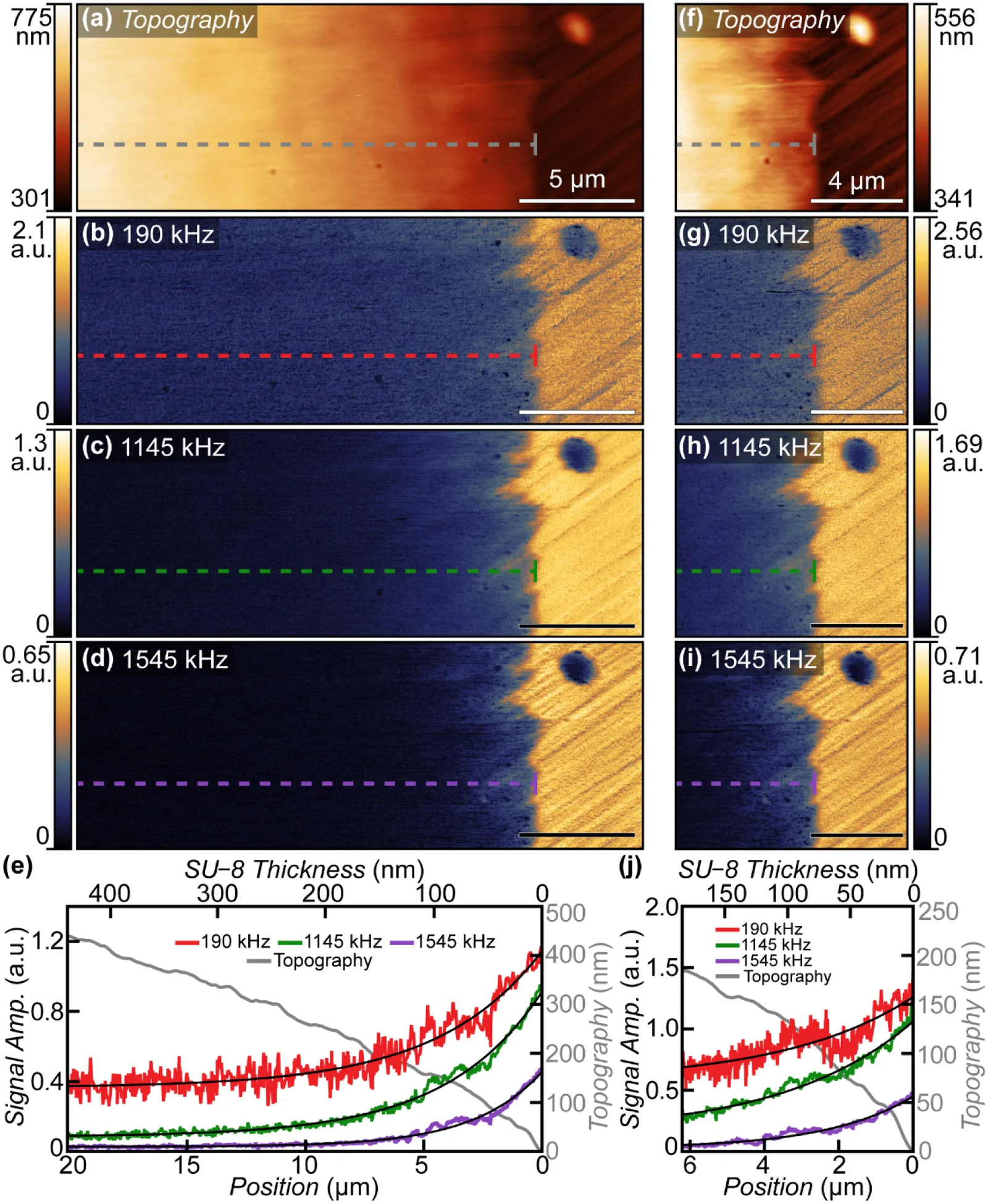
Higher-pixel-resolution, resonance-enhanced, p-polarized AFM-IR images at 1730 cm^–1^ (IP-Dip2 absorption) across the edge of the SU-8 prism, with increasingly thickness to the left. The SU-8 prism sits atop a nearly uniform layer of IP-Dip2, which extends across the entire field of view and is exposed on the right side of the image. (a, f) Topography maps showing the sloped side of the SU-8 prism with thickness decreasing from a maximum height of about 440 nm above the IP-Dip2 disk. Absorption maps obtained at different laser repetition rates (190 kHz, 1145 kHz, and 1545 kHz) with pixel resolution of 23.9 nm (b–d) and 9.8 nm (g–i). (e, j) Color-coded AFM-IR intensity profiles at 1730 cm^–1^ as a function of the local SU-8 thickness obtained from a portion of the sample along the marked with dotted lines in (a–d) and (f–h), respectively. Exponential decay functions, using eq 3, fit to each profile, are shown in black. Gray lines represent the SU-8 thickness along the paths indicated in panels (a) and (f), respectively. Scale bars are 5 μm in (a–d) and 4 μm in (f–i).

Several factors could, in principle, affect the AFM-IR signal transduction and image contrast. First, given the top-side illumination (at 20° incident angle) and 1730 cm^–1^ excitation, the additional interface of the top, non-absorbing SU-8 layer could increase reflection losses, which would be independent of SU-8 thickness, thereby lowering absorption in the buried IP-Dip2 layer in the stratified sample. However, two materials have similar refractive indexes^53,54^ with estimated reflection losses <1%. Second, we can exclude major contributions from acoustic waves, due to acoustic absorption losses in the sample due to the small sample size and typical acoustic attenuation in polymers (≈10^–8^ Np/nm).^55^ Similarly, acoustic reflections losses at the IP-Dip2/SU-8 interface are expected to be small (≈1%) due to the small impedance (*Z* = *ρ*·*v*_l_) mismatch between the two polymers, where *ρ* is the density and *v*_l_ is the longitudinal sound velocity. Third, the variation of the mechanical *Q* factors, which are not compensated for by the PLL, could also result in differences in transduction efficiency and produce contrast unrelated to the sample composition. Table S1 and Figure S6 show that the *Q* factors on the SU-8 portion of the sample generally increase by ≈10% to ≈50% with respect to the exposed IP-Dip2 region. Therefore, for equal light absorption in the IP-Dip2 disk, based on the *Q*-factor alone, a stronger signal is expected from region covered by SU-8, which is contrary to the observations presented in Figures 3 and 4. Therefore, variations of *Q* factors are not responsible for the observed contrast and, in absence of such effects, the contrast in Figures 3 and 4 would be higher. Fourth, with p-polarization, near-field enhancement can boost the signal coming from absorbing materials within ≈50 nm from the top surface (nearest the metal-coated probe tip). Such enhancement is characterized by rapid exponential decay away from the probe tip and is stronger near its apex. Therefore, while a near-field absorption boost is expected for the bare IP-Dip2 portion of the sample, the enhancement should decay very quickly on SU-8 given the exponential near-field decay and because of the enhancement thwarting provided by even the thinnest SU-8 regions where the near-field is the strongest; see schematic illustration in Figure S7.

The data in Figure 3f–i and Figure 4b–d, g–i appear qualitatively similar to the trends observed in a previous report on a PS wedge patterned on a uniform (≈18 μm thick) PMMA layer on a Si substrate measured with p-polarization.^21,47^ In that work, the authors provided an empirical exponential relationship (with 3 fitting parameters) to model the decaying signal intensity (*A*) from the bottom (PMMA) layer as a function of the top (PS) layer thickness (*h*):

3 $$A\left(h\right)=m_{1}+m_{2}\times e^{\frac{-h}{h_{0}}}$$

and *h*_0_ is the distance through the top, non-absorbing SU-8 layer for which the measured signal intensity (due to the absorption in the bottom IP-Dip2 layer) decreases to 1/*e* (i.e., to ≈37% with respect to the signal intensity of the exposed IP-Dip2 region. Importantly, *h*_0_ should not be interpreted as equivalent to depth sensitivity limit, as the measurement retains ≈37% signal intensity at this point, but just a parameter describing the rate of the exponential decay (see also the depth sensitivity discussion, below). We note the bottom PMMA layer (≈18 μm thick) in the sample considered by Dazzi et al.^47^ has a very long thermalization time. Although the 1D diffusion model underlying eq 2 is not rigorously descriptive of such thermally thick samples,^11^ to a first-order approximation the thermalization of an 18 μm thick PMMA layer is estimated to be >1300 μs via eq 2. Consequently, heat accumulation and a steady increase in sample temperature is likely during measurements of that sample even with relatively low laser repetition rates (e.g., 10 kHz, corresponding to 100 μs pulse spacing). In other words, such thick layer of PMMA limits heat dissipation, reducing the influence of the underlaying Si substrate on the measurements. In contrast, given the shorter thermalization time (see below) of the thinner IP-Dip2 layer analyzed here, the BaF_2_ substrate functions as an effective heat sink. Considering the thermal properties of SU-8,^12^ IP-Dip2 ^10^ and BaF_2_ (Table S2), and assuming an interfacial thermal conductance with the BaF_2_ substrate of 3 MW·m^–2^·K^–1^, the thermalization time for the ≈320 nm thick IP-Dip2 is *τ*_IP‑Dip2_ ≈ 460 ns (via eq 1). For *G*_∞_, *τ*_IP‑Dip2_ ≈ 280 ns (via eq 2). This thermalization time scale means that the heating and expansion of the IP-Dip2 layer should be ≈95% dissipated (≈3τ) after 1.38 μs (or ≈0.84 μs in the limit of *G*_∞_) and ≈99.3% dissipated (≈5τ) after 2.3 μs (or ≈1.4 μs in the limit of *G*_∞_). By comparison, the temporal spacing of the laser pulses used here is ≈0.65 μs at 1545 kHz, ≈0.87 μs at 1145 kHz, ≈2.56 μs at 391 kHz, and ≈5.26 μs at 190 kHz, respectively. Therefore, for our sample, we expect the effect of heat accumulation and of the laser frequency to be negligible for the data recorded at 190 kHz and 391 kHz.

By fitting the same empirical relationship proposed by Dazzi and coworkers (eq 3) to our measurements, we can independently estimate *h*_0_ for different laser repetition rates. The fitted curves are overlaid on the absorption profiles in Figure 4e, j, with the complete set of fitting parameters summarized in Table S3 Most significantly, for p-polarization, the *h*_0_ characterizing absorption intensity decay is found to be ≈(76±2.6) nm, ≈(75±1.4) nm, and ≈(55±1.1) nm at repetition rates of 190 kHz, 1145 kHz, and 1545 kHz, respectively. Uncertainties in *h*_0_ represent a single standard deviation derived from the exponential fit covariance matrix, which are generally smaller than the uncertainty in the SU-8 thickness (3.8 nm). The latter uncertainty corresponds to a single standard deviation of the propagated uncertainties obtained by measuring the thickness of the exposed IP-Dip2 and SU-8 regions with the AFM. We note, however, that the lower signal-to-noise ratios in our measurements and the larger sample roughness, relative to the profiles reported by Dazzi et al., results in greater uncertainties in the estimated local thickness and in the fitting parameters to our data (see Table S3). Nevertheless, we observe a similar trend in *h*_0_, with generally smaller *h*_0_ (faster signal decay) at increased pulse frequencies, in agreement with Dazzi et al.’s report. However, at the lowest tested frequency (190 kHz), the marginal increase *h*_0_ is inconsistent with the trend rate. This anomaly may be due to the lower signal-to-noise ratio in the measured absorption profile and to the absence of heat accumulation in our sample due to its fast thermalization compared to the laser pulse spacing at this frequency.

## Conclusion

In summary, here, we elucidate the effects of sample stratification on the intensity of detected AFM-IR absorption signal using a heterogeneous, 3D-printed polymer structure consisting of an IP-Dip2 disk partially covered by an SU-8 prism. This structure, engineered to have a top layer (SU-8) with an approximately linearly varying thickness covering a nearly uniform bottom layer (IP-Dip2), enables us to systematically discern the effects of detection frequency on the detected AFM-IR signal for stratified samples. Notably, the signal from the top (SU-8) absorbing layer showed a nearly linear monotonic increase with increasing SU-8 thickness for both on- and off-resonance detection schemes, independently from the cantilever detection frequency, corroborating reported results for homogeneous, non-stratified, samples.^10,15,49^

For off-resonance excitations, the AFM-IR signal intensity due to absorption in the bottom (IP-Dip2) layer was found to depend significantly on the cantilever detection frequency, which can either amplify, reduce, or not affect the signal intensity. Therefore, to map the composition of the bottom layer in stratified samples with off-resonance AFM-IR measurements, we suggest selecting an *ad hoc* detection frequency that minimizes the effects of material-specific, near-surface, tip-sample interactions.

In contrast, for on-resonance excitations, the AFM-IR signal intensity due to absorption in the bottom (IP-Dip2) layer was attenuated by the SU-8 top layer for all the measured frequencies and for both s- and p-polarizations. The attenuation of the signal intensity of the bottom layer displays an approximately exponentially decreasing trend as a function of the non-absorbing, top-layer (SU-8) thickness, similarly to a recent report.^47^ Here, we tested a variety of factors that affect the signal intensities. Contrast due to material-specific damping, related to mechanical *Q* factors and the mechanical gain of the AFM cantilever, cannot explain the exponential signal attenuation since *Q* factors are larger on SU-8 than on IP-Dip2. We attribute the observed signal attenuation to a combination of short-range (≈50 nm), frequency-independent, tip-enhancement effects, which are difficult to quantify, and longer range thermal modulation depth, which was reported to be inversely proportional to the detection frequency in a recent publication.^47^ Our data do not show a clear trend linking the ≈63% signal decay (1/*e*) to the detection frequency, possibly because the BaF_2_ substrate acts as a heat sink and prevents heat accumulation in the sample at relatively low frequencies (less than about 400 kHz to 700 kHz). For the resonant excitation scheme, an exponential decay function captures the general experimental trend of the signal intensity in a stratified sample reasonably well. However, the ≈63% signal intensity decay point (*h*_0_) should not be interpreted as equivalent to depth sensitivity limit. For example, IP-Dip2 absorption was detected in all our images even under the maximum SU-8 thickness (440 nm) suggesting a depth sensitivity >440 nm for all the frequencies tested (i.e., up to 1545 kHz).

By revealing the effects of sample stratification on the AFM-IR signal intensities in a variety of conditions, our work is useful to advance AFM-IR toward more quantitative chemical compositional imaging at the nanoscale. A very simple, nearly assumption-free, sample thermalization model (eq 1) is sufficient to qualitatively rationalize the observed signal trends. A main corollary of our work is that the AFM-IR probed depth is not an intrinsic (i.e., exclusive) characteristic of the measurement parameters (e.g., excitation frequency) but also depends on the extent that heat accumulates in the sample, as a function of the sample the thermalization time. Therefore, future work shall consider the interplay of the sample thermalization time, pulse spacing, and the effect of the substrate for measuring and analyzing stratified samples, particularly if attempting depth reconstructions. This work also highlights the need for further detailed modeling to fully describe the AFM-IR signal quantitatively as many factors can contribute to absolute AFM-IR signal intensity, as discussed previously.^4^ As more studies are directed toward quantitative characterizations of the composition in stratified samples, adopting a common practical definition of the probed depth will facilitate comparisons. We suggest using the 1/3*e* (≈95%, i.e., 3*h*_0_) signal decay, rather than 1/*e* (63% decay), as a convention for referring to AFM-IR depth sensitivity in practice.

## Supplementary Material



## Footnotes

1 The full description of the procedures used in this paper requires the identification of certain commercial products and their supplier. The inclusion of such information should in no way be construed as indicating that such products are endorsed by NIST or are recommended by NIST or that they are necessarily the best materials for the purposes described.

## Notes

Off-resonance AFM-IR signal amplitudes and frequency response as a function of SU-8 thickness; off-resonance AFM-IR maps; on-resonance AFM-IR maps; quality factor analysis; schematic of near-field enhancement; material properties; and fitting parameters for eq 3 (PDF)
