# Supplementary material for: AFM-IR Depth Sensitivity on Vertically Stratified Polymer Structures
Source: Anal Chem. 2026 Sep 7;98(36):26410–20. doi: 10.1021/acs.analchem.6c01324 (PMC13584627; doi:10.1021/acs.analchem.6c01324)
Supplement: Supplementary file 1 [file ac6c01324_si_001.pdf]

Supporting Information:

AFM-IR Depth Sensitivity on Vertically Stratified Polymer Structures

Devon S. Jakob,[1] Jeffrey J. Schwartz,[2,3], Karen E. Grutter,[2,3] and Andrea Centrone[1,†]

[1]Nanoscale Devices Characterization Division, Physical Measurement Laboratory, National Institute of Standards and Technology, Gaithersburg, Maryland 20899, United States

[2]Laboratory for Physical Sciences, College Park, Maryland 20740, United States

[3]Department of Electrical and Computer Engineering, University of Maryland, College Park, Maryland 20742, United States

[†]Corresponding author email: andrea.centrone@nist.gov

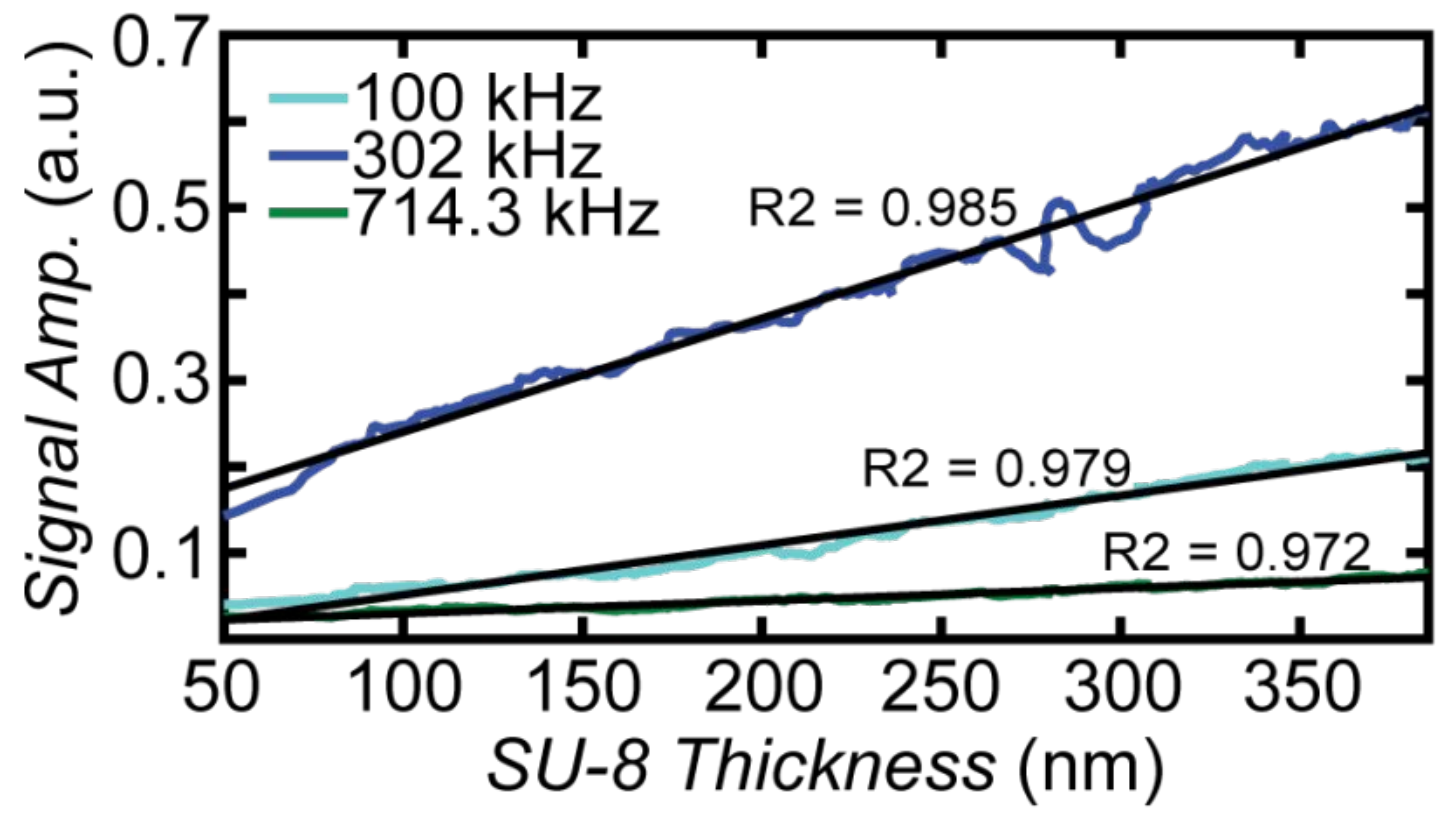

Figure S1: Off-resonance AFM-IR signal amplitudes as a function of SU-8 thickness, obtained at 1510 cm^{-1}, corresponding to the maps in Figure 2a-c. Linear fits reveal a nearly linear monotonic in signal intensity for all cantilever detection frequencies (R^2 = 0.972 – 0.985) over the displayed thickness range, i.e., from 50 nm to 385 nm.

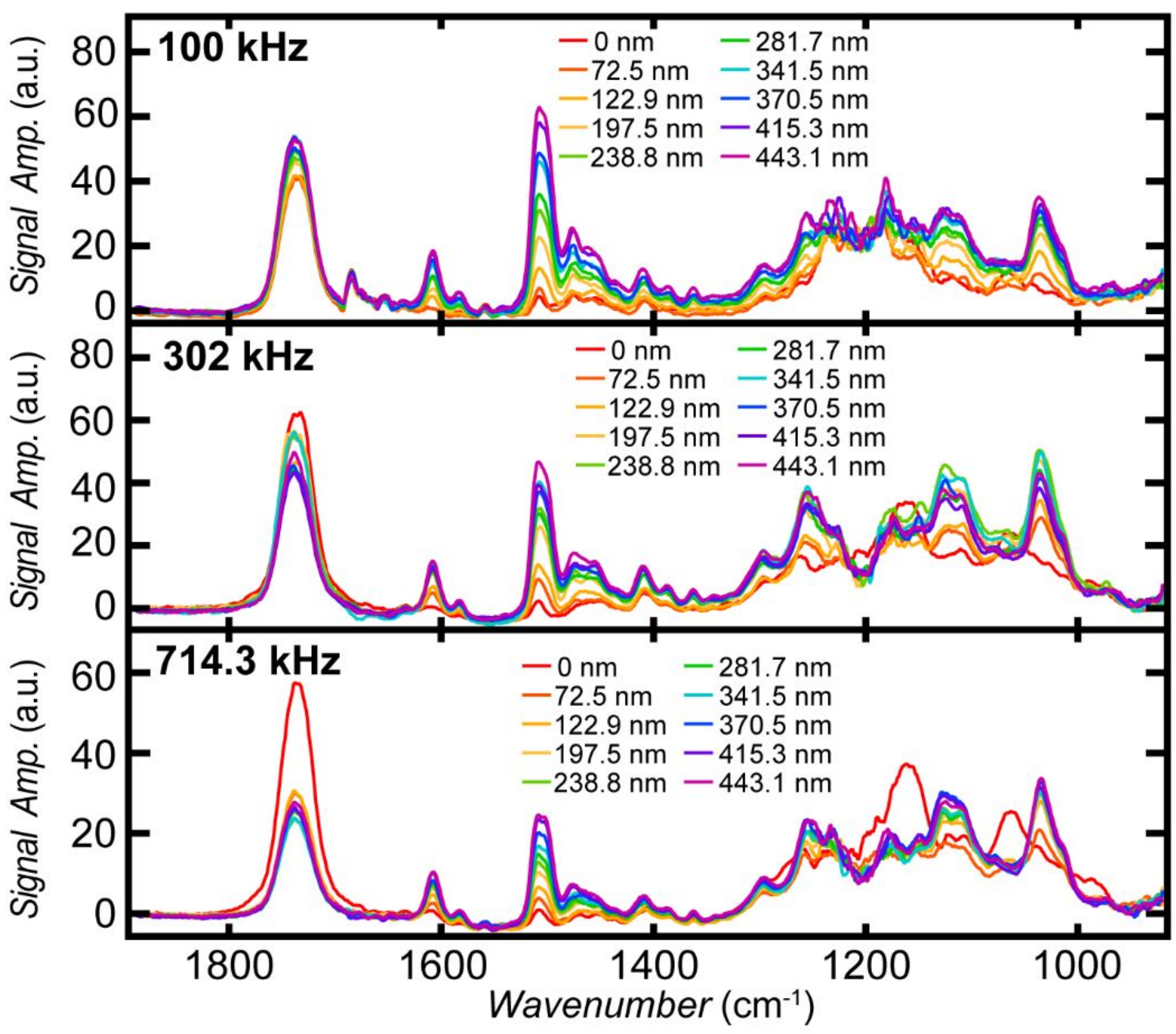

Figure S2: Off-resonance AFM-IR spectra obtained in correspondence of different SU-8 thickness, at 3 laser repetition rates corresponding to the images in Figure 2 of the main text. Spectra were obtained at a rate of 50 cm^{-1}/s and with a resolution of 1 cm^{-1}. 10 spectra were averaged at each location and a Savitzky-Golay filter with a third-order polynomial was used to smooth the spectra prior to baseline-fitting. All measurements were obtained with a ≈ 10 % laser duty cycle with s-polarization.

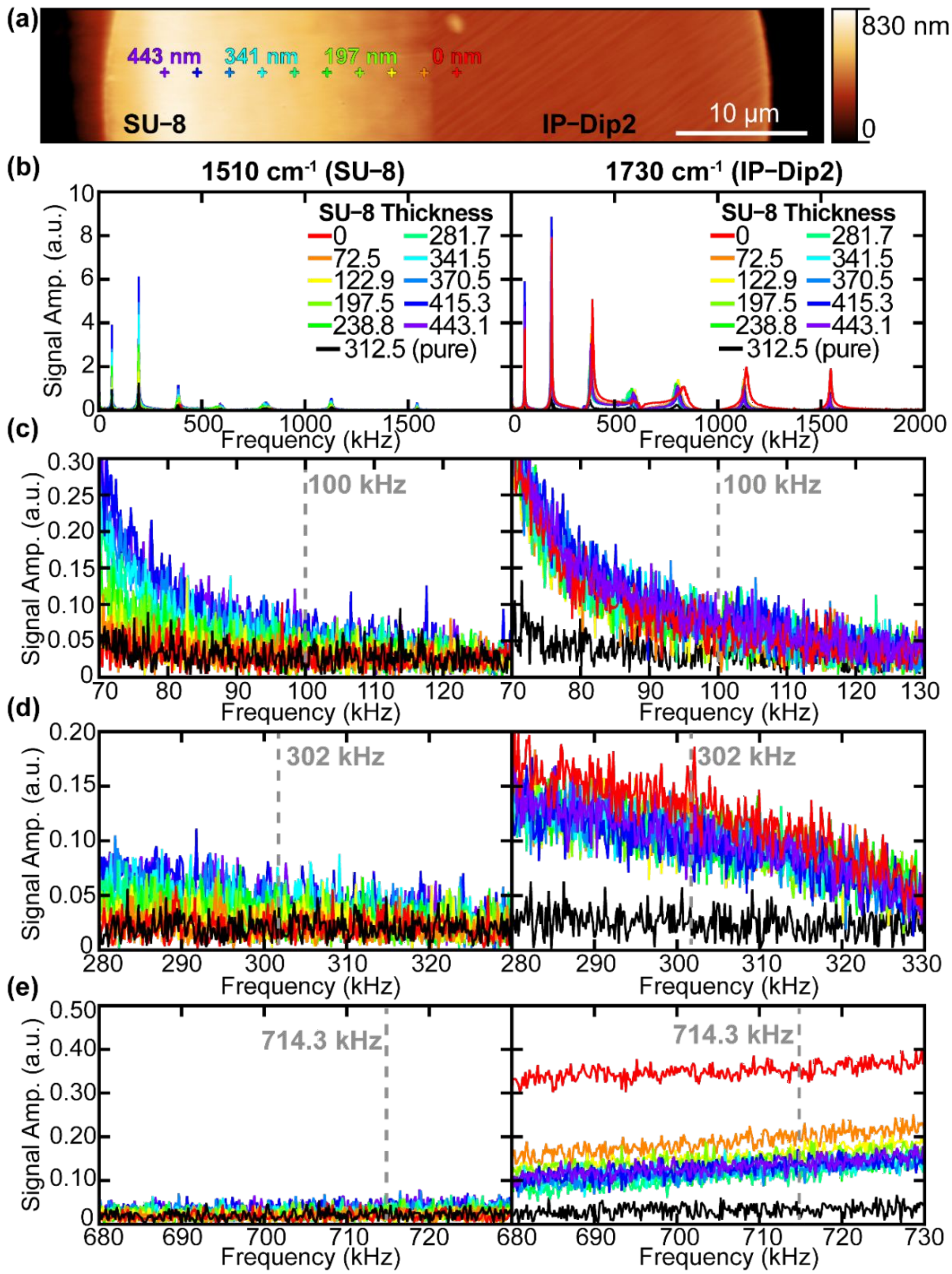

Figure S3: **(a)** Topography of the sample consisting of a SU-8 prism fabricated on top of an IP-Dip2 disk on a BaF_2 substrate. **(b)** Frequency-domain AFM-IR cantilever excitation amplitudes, measured at the color-coded locations indicated in (a) by sweeping the laser pulse repetition rate while illuminating at the characteristic absorption frequencies of SU-8 (1510 cm^{-1}, left) and IP-Dip2 (1730 cm^{-1}, right). The black trace is the absorption signal measured on a structure composed only of SU-8 (i.e., no IP-Dip2). **(c–e)** Magnified views of the spectra shown in (b), detailing the cantilever transduction at repetition rates at 100 kHz, 302 kHz, 714 kHz, corresponding to the off-resonant images in Figure 2.

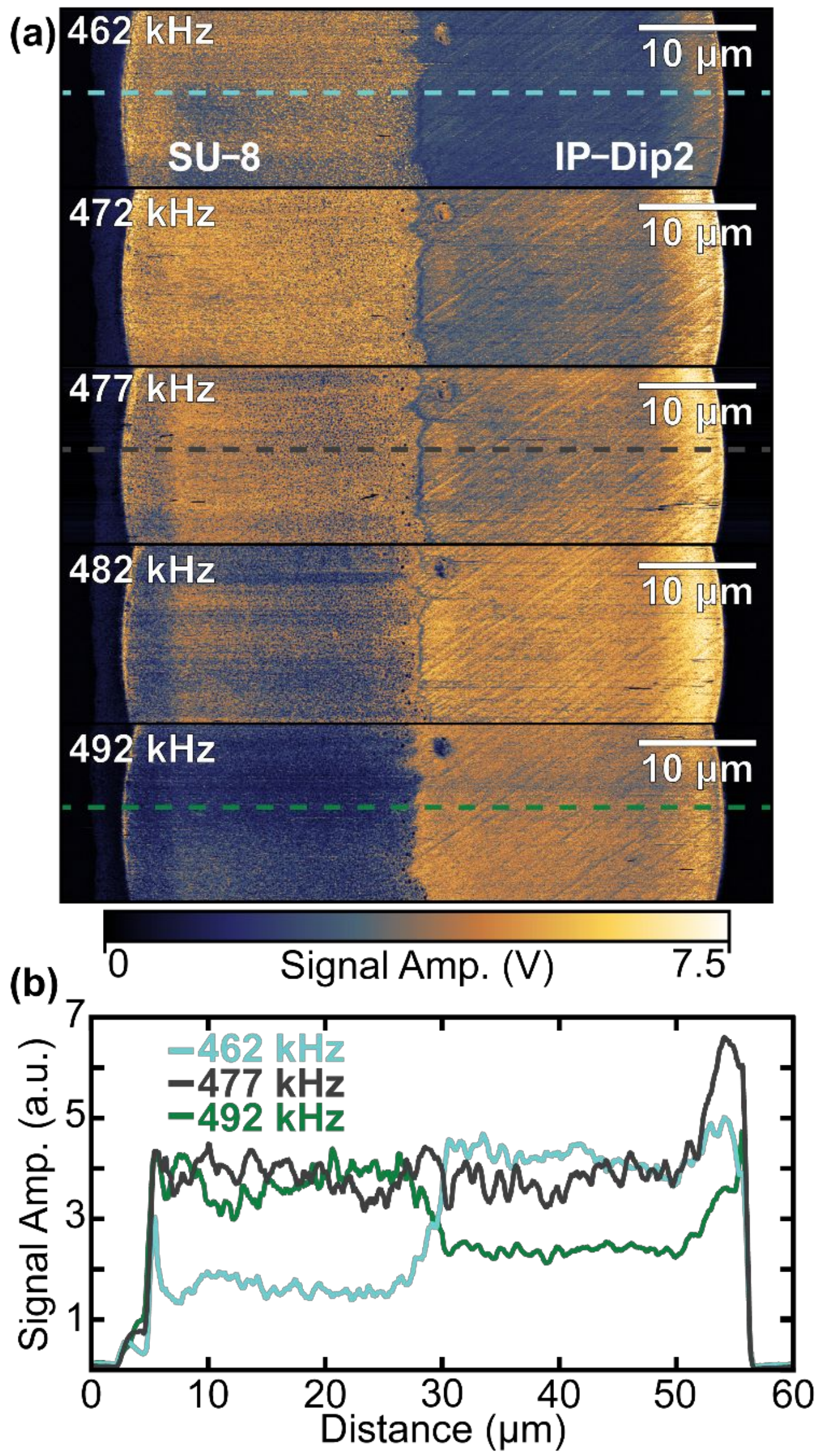

Figure S4: (a) Off-resonance AFM-IR absorption maps (≈ 10 % laser pulse duty cycle, s-polarization, analogous to Figure 2e–h) obtained at 1730 cm^{-1} using a different AFM probe. By adjusting the laser repetition rate by only a few kHz (from 462 kHz to 492 kHz), the signal contrast between the SU-8/IP-Dip2 region (left) and the bare IP-Dip2 region (right) can be reversed (e.g., compare images at 462 kHz and 492 kHz), or approximately negated (at 477 kHz). **(b)** Absorption signal profiles, measured at different repetition rates, across the dashed paths indicated in (a).

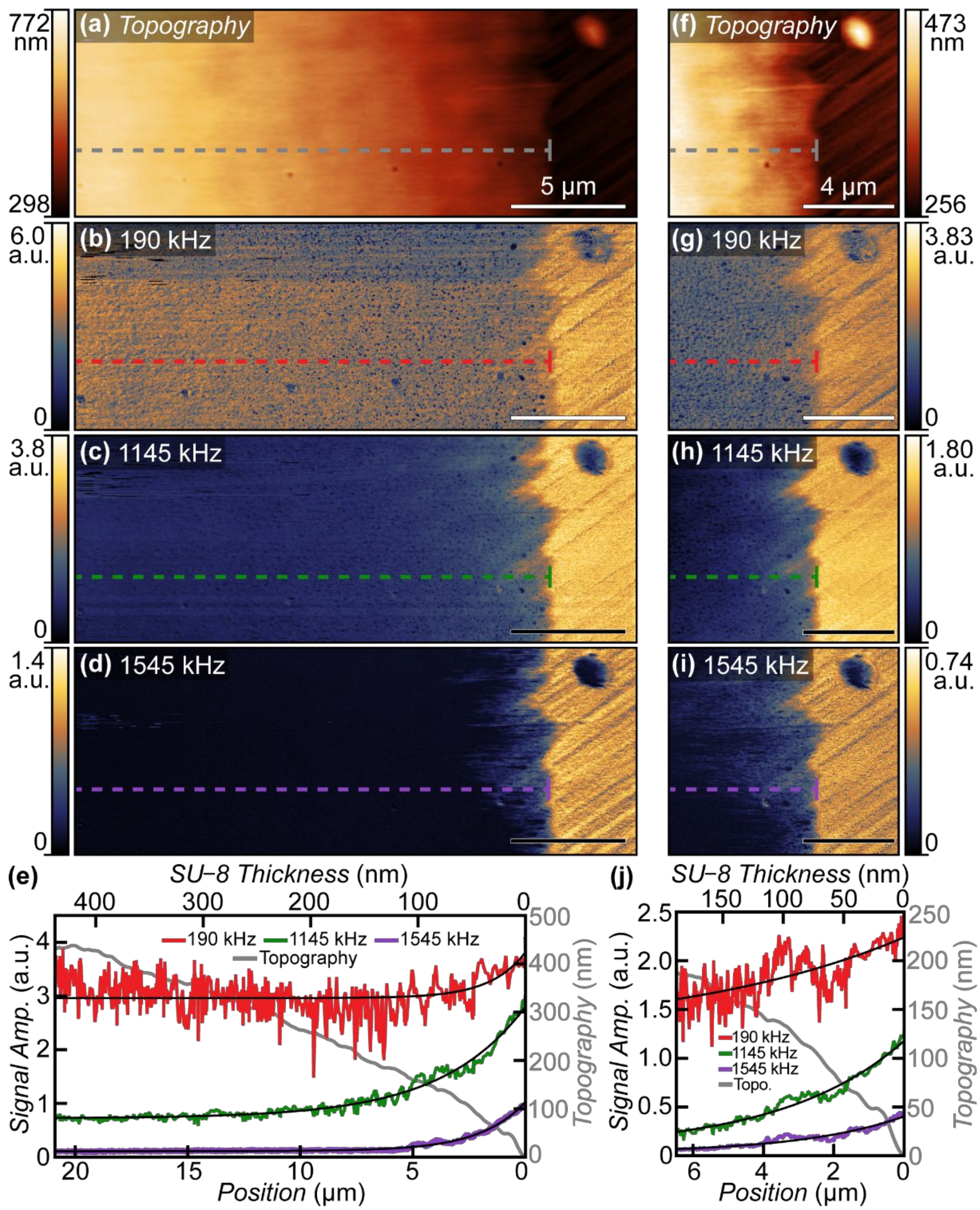

Figure S5: Resonance-enhanced s-polarized AFM-IR images at 1730 cm^{1} (IP-Dip2) around the boundary between the SU-8 prism (left) and IP-Dip2 disk (right). **(a)** Topography map, showing the sloped side of the SU-8 prism with thickness decreasing from ≈ 440 nm above the IP-Dip2 disk. **(b–d)** Absorption maps (23.9 nm/pixel resolution) obtained at different laser repetition rates. **(f)** Topography map (9.8 nm/pixel) near the edge of the SU-8 prism. **(g–i)** Absorption maps obtained at different laser repetition rates over the same region shown in (a). **(e, j)** Color-coded AFM-IR intensity profiles at 1730 cm^{-1} as a function of the local SU-8 thickness obtained from a portion of the sample along the marked with dotted lines in (a–d) and (f–h) respectively. Fitted

exponential decay functions, Eq. 2, to each profile, are shown in black. Gray lines represent the SU-8 thickness along the paths indicated in panels (a) and (f), respectively.

Quality Factor Determination

Contact-mode resonance quality (*Q*) factors of the AFM probe were determined by illuminating the sample at constant wavelength (either 1510 cm^{-1} or 1730 cm^{-1}) while sweeping the laser repetition rate from 0 kHz to 2000 kHz. (see Figure S1b). The experiments were repeated after positioning the probe on sample at positions with different, known SU-8 thicknesses. Lorentzian functions were fit to the data using an iterative damped least-squares method (Levenberg-Marquardt algorithm) after partitioning each curve into sections that span the tested range of the probe's mechanical resonance (e.g., at ≈ 190 kHz, ≈ 391 kHz, ≈ 1145 kHz, and ≈ 1545 kHz). Uncertainties are computed from one standard deviation of the peak center and half-width at half-maximum found through the Lorentzian fit. The effect of the *Q* factor is found as the percent difference between the highest and lowest calculated *Q* factor for each set.

Table S1: Quality factors of the AFM probe used in this experiment, obtained by fitting a Lorentzian function to the laser pulse frequency amplitude response curves (shown in Figure S1) at sample locations characterized by different SU-8 thicknesses.

SU-8 Thickness (nm)	Resonance Quality Factor, *Q*							
	190 kHz		391 kHz		1145 kHz		1545 kHz	
	1510 cm^{-1}	1730 cm^{-1}	1510 cm^{-1}	1730 cm^{-1}	1510 cm^{-1}	1730 cm^{-1}	1510 cm^{-1}	1730 cm^{-1}
0	57.4 ± 1.59	50.0 ± 1.18	43.1 ± 4.01	36.3 ± 3.34	44.2 ± 14.5	35.7 ± 3.28	145 ± 49.8	99.2 ± 5.92
72.50	57.7 ± 1.44	55.8 ± 1.21	42.2 ± 3.33	40.0 ± 3.21	67.6 ± 9.84	56.6 ± 3.52	186 ± 33.1	141 ± 6.14
122.9	65.0 ± 1.48	62.5 ± 1.09	42.5 ± 4.07	49.0 ± 3.49	85.4 ± 9.40	70.2 ± 3.84	242 ± 28.1	151 ± 6.09
197.5	61.8 ± 1.34	61.2 ± 1.20	43.8 ± 2.64	41.9 ± 3.28	67.3 ± 5.98	67.5 ± 3.81	186 ± 13.8	151 ± 5.92
238.7	54.3 ± 1.32	56.2 ± 1.19	37.2 ± 4.16	38.3 ± 3.51	100 ± 5.80	79.6 ± 4.38	226 ± 19.9	176 ± 5.76
443.1	64.1 ± 1.35	56.1 ± 1.25	45.3 ± 2.65	40.7 ± 3.09	77.7 ± 4.39	70.9 ± 3.85	185 ± 8.62	165 ± 6.31
**% difference**[a]	11.6	20.0	17.8	25.8	56.0	55.1	40.3	43.5

[a]Computed as the percent difference in the maximum and minimum quality factors obtained within each data set.

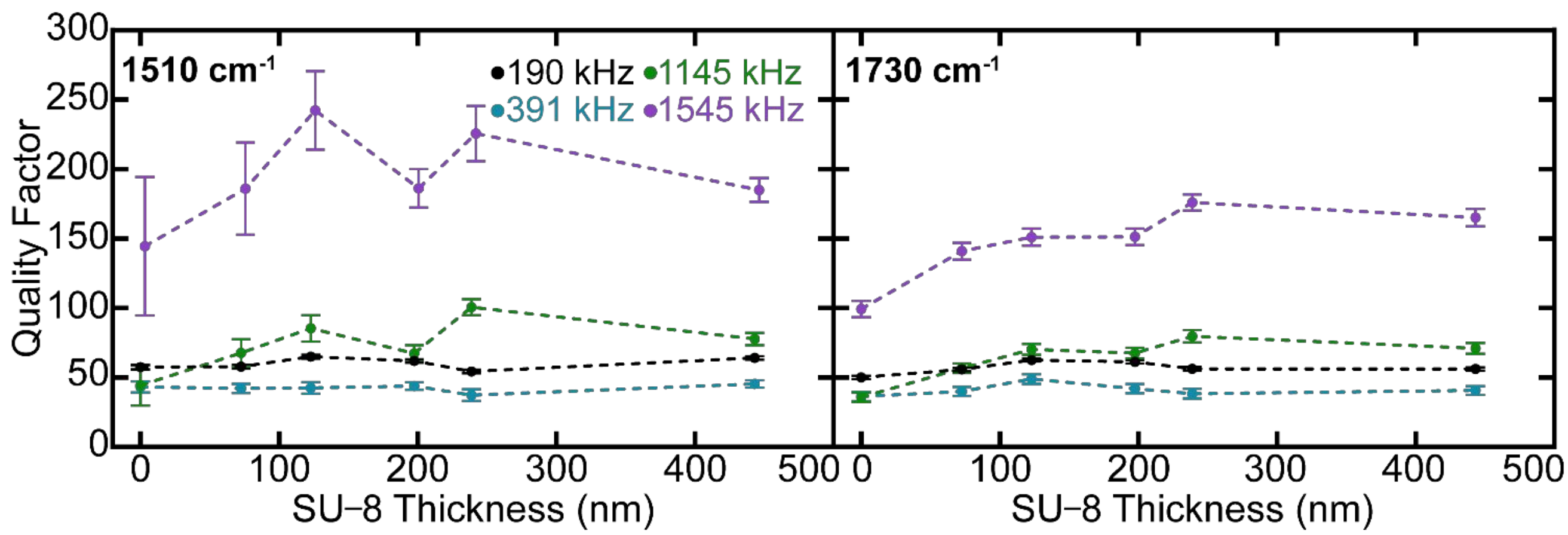

Figure S6: Quality (*Q*) factors of the AFM cantilever, in contact with the SU-8/IP-Dip2 sample at different SU-8 thicknesses, while illuminating the sample at 1510 cm^{-1} (SU-8 absorption, left) and at 1730 cm^{-1} (IP-Dip2 absorption, right).

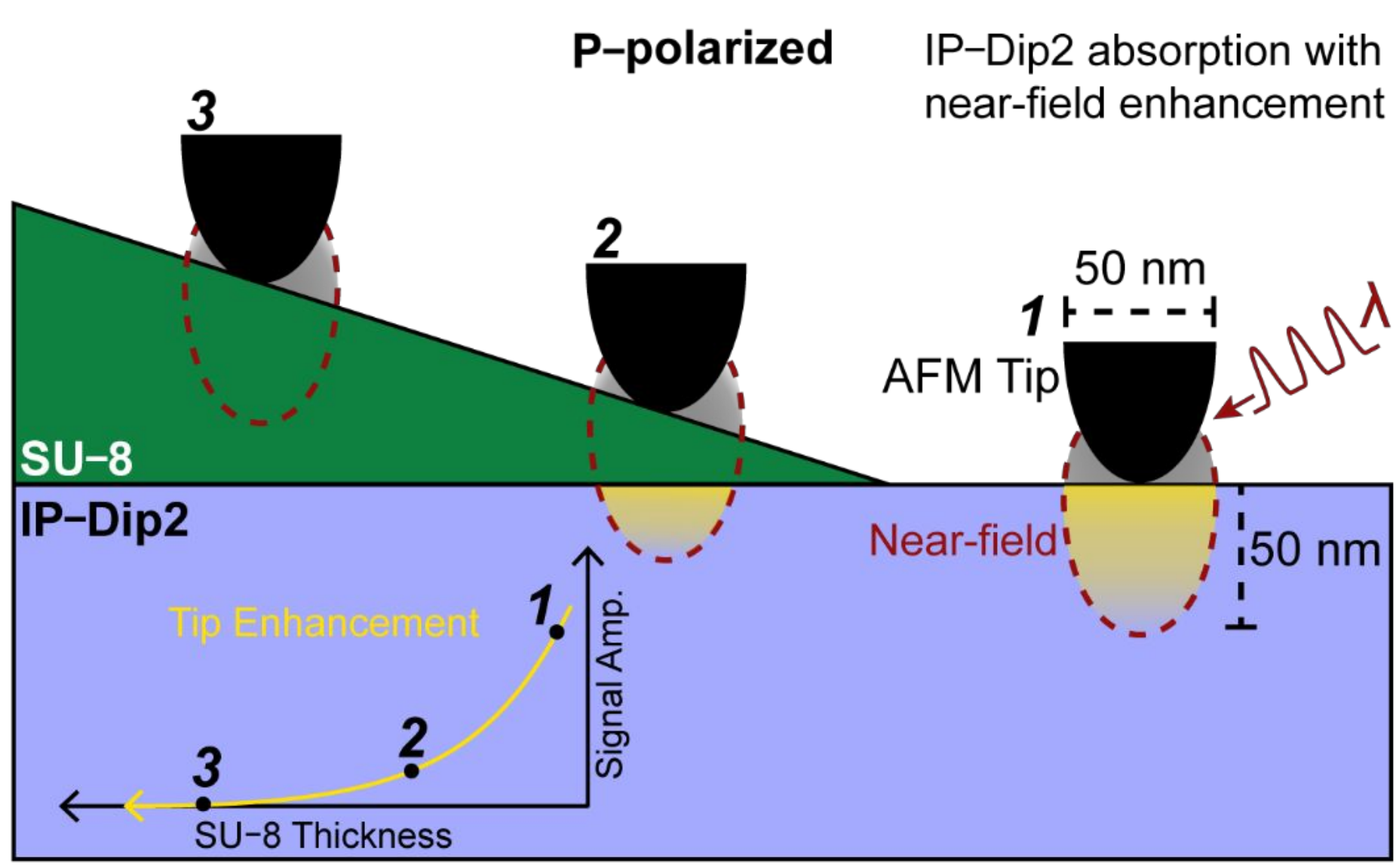

Figure S7: Illustration of light absorption with p-polarized light in IP-Dip2 (blue) with the region subject to near-field enhancement indicated in yellow. The enhancement boosts the signal amplitude for the fraction of absorbing sample near the tip within ≈ 50 nm of the sample surface (Point 1, right, comparable to the AFM tip diameter). As the tip moves over the region covered by SU-8 (green), the effect of the near-field enhancement decreases significantly with the first few nanometers of the non-absorbing SU-8 layer (Point 2, middle), When the SU-8 thickness exceeds the depth for the tip-enhanced near-field, no significant absorption enhancement occurs in the IP-Dip2 layer (Point 3, left).

Table S2: Thermal properties of IP-Dip2 and BaF_2.

Property	Material	
	IP-Dip2	BaF_2[1]
Thermal Conductivity, *k* (W/m·K)	0.28[2]	11.72
Specific Heat, c_p (J/kg·K)	1500[2]	410
Density, ρ (kg/m^3)	1260[3]	4890
Interfacial Thermal Conductance IP-Dip2/BaF_2 (MW·m^{-2}·K^{-1})	3	

Table S3: Parameters representing the best fit of Equation 3 to the IP-Dip2 absorption profiles shown in Figure 4e,j, for p-polarization. Uncertainties represent a single standard deviation derived from the fit covariance matrix.

Frequency (kHz)	Fit Parameters		
	m_1	m_2	h_0 (nm)
190	0.372 ± 0.005	0.74 ± 0.01	76 ± 2.6
1145	0.082 ± 0.003	0.74 ± 0.01	75 ± 1.4
1545	0.025 ± 0.001	0.352 ± 0.004	55 ± 1.1

References

1. Crystran Ltd. Barium Fluoride (BaF_2). https://www.crystran.com/optical-materials/barium-fluoride-baf2/ (accessed December 8th 2025).
2. Jakob, D. S.; Schwartz, J. J.; Pavlidis, G.; Grutter, K. E.; Centrone, A., Understanding AFM-IR Signal Dependence on Sample Thickness and Laser Excitation: Experimental and Theoretical Insights, *Anal. Chem.* **2024,** *96*, 41, 16195-16202.
3. Nanoscribe GmbH IP-Dip2 Mass Density. https://support.nanoscribe.com/hc/en-gb/articles/360017836760-IP-Dip2 (accessed December 8th 2025).
